# Crotoxin Conjugated to SBA-15 Nanostructured Mesoporous Silica Induces Long-Last Analgesic Effect in the Neuropathic Pain Model in Mice

**DOI:** 10.3390/toxins11120679

**Published:** 2019-11-20

**Authors:** Morena Brazil Sant’Anna, Flavia Souza Ribeiro Lopes, Louise Faggionato Kimura, Aline Carolina Giardini, Osvaldo Augusto Sant’Anna, Gisele Picolo

**Affiliations:** 1 Special Laboratory of Pain and Signalling, Butantan Institute, São Paulo 05503-900, Brazil; morena.santanna@butantan.gov.br (M.B.S.); flavia.lopes@butantan.gov.br (F.S.R.L.); louise.vieira@butantan.gov.br (L.F.K.); aline.giardini@butantan.gov.br (A.C.G.); 2 Laboratory of Immunochemistry, Butantan Institute, São Paulo 05503-900, Brazil; gbrazil@usp.br

**Keywords:** neuropathic pain 1, crotoxin 2, SBA-15 nanostructured silica 3, partial sciatic nerve ligation 4, analgesia 5, hypernociception 6

## Abstract

Neuropathic pain is a disease caused by structural and functional plasticity in central and peripheral sensory pathways that produce alterations in nociceptive processing. Currently, pharmacological treatment for this condition remains a challenge. Crotoxin (CTX), the main neurotoxin of *Crotalus durissus terrificus* rattlesnake venom, has well described prolonged anti-inflammatory and antinociceptive activities. In spite of its potential benefits, the toxicity of CTX remains a limiting factor for its use. SBA-15 is an inert nanostructured mesoporous silica that, when used as a vehicle, may reduce toxicity and potentiate the activity of different compounds. Based on this, we propose to conjugate crotoxin with SBA-15 (CTX:SBA-15) in order to investigate if when adsorbed to silica, CTX would have its toxicity reduced and its analgesic effect enhanced in neuropathic pain induced by the partial sciatic nerve ligation (PSNL) model. SBA-15 enabled an increase of 35% of CTX dosage. Treatment with CTX:SBA-15 induced a long-lasting reduction of mechanical hypernociception, without modifying the previously known pathways involved in antinociception. Moreover, CTX:SBA-15 reduced IL-6 and increased IL-10 levels in the spinal cord. Surprisingly, the antinociceptive effect of CTX:SBA-15 was also observed after oral administration. These data indicate the potential use of the CTX:SBA-15 complex for neuropathic pain control and corroborates the protective potential of SBA-15.

## 1. Introduction

Chronic pain is a disease that frequently accompanies, but may outlast, inflammatory tissue damage and/or nerve injury. It is generally characterized by its spontaneous firing, hypernociception that is known as an increase of pain elicited by a noxious stimulus, and/or allodynia that is known as a pain elicited by normally innocuous stimuli [1,2].

Simultaneously with these symptoms, patients with chronic pain present lethargy, depression, and anxiety, characterizing a classic systemic disease and suggesting immune activity [3]. This disease not only changes the activity of the neuronal system, but encompasses interactions between neurons and immunocompetent cells, including antigen presenting cells, T lymphocytes, and glial cells, which mediate this interaction through the release of proinflammatory cytokines and chemokines [4,5,6]; this relationship can be understood as a class of pathophysiological pathway that enfolds the neuroimmune interface [3].

Neuropathic pain is widely recognized as one of the most difficult syndromes to be treated, representing a challenge to basic scientific research and clinical practice. Common analgesic therapies which include anti-inflammatory drugs and opioids have often little or no effect, however non-conventional painkillers such as anti-epileptics or antidepressants (as serotonin reuptake inhibitors, for example) can be effective [7]. Currently, new drugs are in development, like the G protein-coupled receptors (GPCR) ligands which include α2 adrenergic, cannabinoids, and acetylcholine receptors [8,9]. In spite of this, many patients continue to suffer from moderate to severe pain when taking prescribed medications for their condition [10] or have to deal with serious side effects. Therefore, the development of new drugs for the treatment of neuropathic pain is still an urgent need.

The modulatory effect of the crude venom from the South American rattlesnake *Crotalus durissus terrificus* over different conditions has been an object of several previous studies [11,12,13]. Crotoxin (CTX) is the main toxin responsible for the high toxicity of this venom [14]. In low doses, CTX presents immunomodulatory, anti-inflammatory, antitumor, and antinociceptive effects [13,15,16,17,18]. Regarding antinociception, studies have shown that CTX can inhibit both acute [19,20] and chronic pain when topically applied at the site of the nerve injury [16]; this effect is mediated by central muscarinic receptors, α-adrenergic receptors, serotonergic and noradrenergic systems [16,19]. In addition, studies have demonstrated that lipid mediators derived from the lipoxygenase pathway are involved in the anti-inflammatory effect of CTX, as an increase of lipoxin A4 production can be observed by macrophages in culture, which occurs through an interaction with G protein-coupled receptors, that belong to the formyl peptide receptor family [13,16,21].

The mesoporous silica nanoparticles (MSNs) are ordered porous material that, due to their physicochemical and structural properties, can be used as an efficient vehicle/adjuvant, acting as drug delivery systems [22,23]. The control of different parameters during MSNs synthesis such as pore size and volume, morphology and particle size turn them into a versatile vehicle because they can load from small to macromolecules [24]. Due to the ability of controlling their surface aspect during the synthesis and their controlled drug release property, they are widely explored as a means to raise site-specific delivery of drugs and avoid side effects, modifying the drug loading potential and reducing the compounds toxicity [24,25,26].

Thus, the aim of this study was to evaluate whether the nanostructured SBA-15 silica can improve the antinociceptive effect of CTX on the neuropathic pain model induced by PSNL and/or protect the organism from CTX toxicity. It was demonstrated that the SBA-15 silica both enables the increase of the therapeutic dose as well as prolongs the antinociceptive effect of CTX without altering the antinociceptive mechanism; furthermore, when conjugated to SBA-15, the oral route becomes a possible path for CTX administration since the antinociceptive effect is preserved.

## 2. Results

### 2.1. CTX LD_50_ Increased When Adsorbed to SBA-15

Firstly, a range of doses were tested to determine the lethal dose 50% (LD50) of CTX and CTX:SBA-15 complex to be used in this study. Unconjugated CTX induced the death of 50% of the animals at the dose of 106 μg/kg (63.2—177.9). Subsequently, an increase of 35% was needed in the CTX dose when complexed to SBA-15 to induce the death of half of the mice in the group, LD50 was found to be 143.2 μg/kg (70.4–291.2) (Table S1). Although this value remained within the confidence interval, there was an increase of 11.4% and 63.7%, respectively, in the lower and upper level values of the confidence limit of this data. Therefore, adsorption to SBA-15 allowed an increase of CTX dosage in comparison to that previously reported [13] (from 40 to 54 μg/kg).

### 2.2. CTX:SBA-15 Prolongs the Antinociceptive Effect of CTX in the PSNL Model 

**Figure 1:**
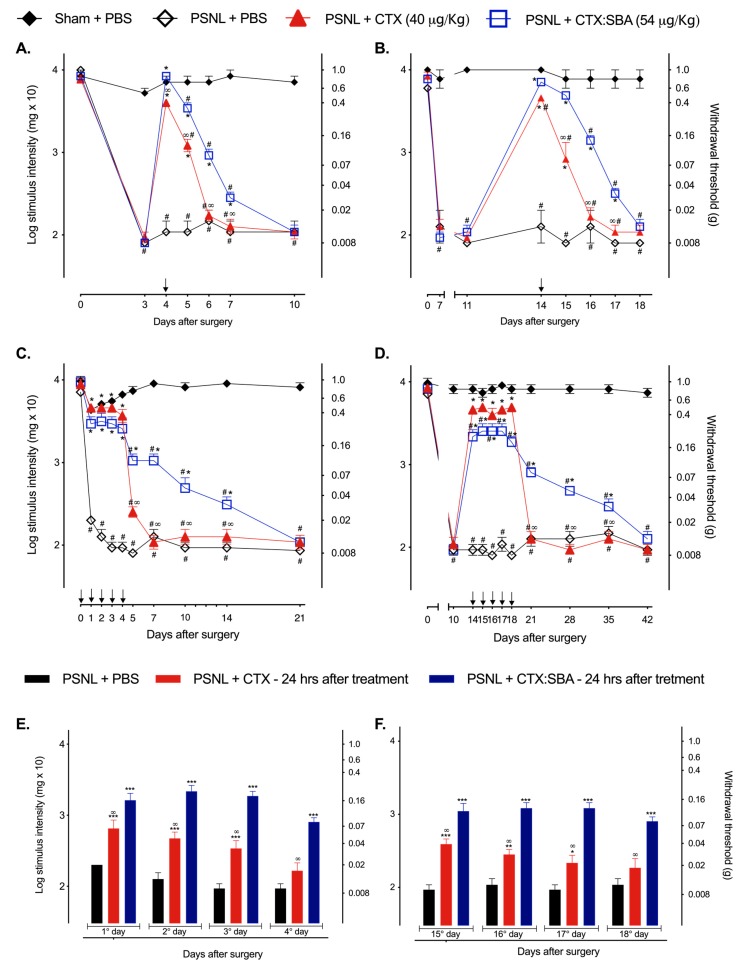
Effects of CTX and CTX:SBA-15 on mechanical hypernociception induced by PSNL. The pain threshold of the animals who underwent surgery (sham and PSNL) was evaluated from day 0 (baseline) to the 10th (**A**), 18th (**B**), 21th (**C**), or to 42th (**D**) days after PSNL. Measurements were performed daily after a single administration of CTX (40 µg/kg, s.c.), CTX:SBA-15 (54 µg/kg, s.c.), or PBS (200µL, s.c.) (**A**,**B**) or 1 h (**C**,**D**) and 24 h (before the following injection) (**E**,**F**) after CTX (40 µg/kg), CTX:SBA-15 (54 µg/kg), or PBS (control) treatment with one (**A**,**B**) or five doses (**C**–**F**). Arrows indicate the days of compounds administration. The nociceptive threshold was assessed using the von Frey filaments. Results are expressed as mean (± SEM) n = 6, representative of three independent experiments. * *p* < 0.05, ** *p* < 0.01, *** *p* < 0.001 indicates significant difference when compared to the PSNL + PBS group. ^#^ *p* < 0.01 indicates significant difference when compared to the Sham + PBS group. ∞ *p* < 0.01 indicates a statistically significant difference when compared to PSNL + CTX:SBA-15 group.

To evaluate the effects of CTX or CTX:SBA-15 complex in the acute and chronic phases of hypernociception induced by PSNL, both treatments were administered in 1 or 5 consecutive doses (1 daily dose for 5 days). As expected, mice submitted to PSNL surgery and treated with PBS showed a significant decrease in the nociceptive threshold when compared to sham + PBS group during all evaluated days (Figure 1A–D). During the treatment period, the animals were evaluated 1 and 24 h after administration of CTX or CTX:SBA-15. To verify whether SBA-15 could modify the nociceptive threshold after surgery, the animals were administered with a single dose of SBA-15 on the 3rd and 14th day after surgery, and no difference was observed in either group (sham and PSNL) (Figure S1). Even though, a single administration with CTX as well as CTX:SBA-15 in both acute (Figure 1A) and chronic (Figure 1B) phases reverted the hypernociception induced by PSNL surgery 1 h after administration; 48 h after treatments, the antinociceptive effect of unconjugated CTX was no longer observed whereas animals treated with CTX:SBA-15 still presented partial reversion of PSNL-induced hypernociception (Figure 1A,B).

When both the CTX and CTX:SBA-15 PSNL group received 5 consecutive daily doses, a complete inhibition of mechanical hypernociception was observed 1 h after each treatment in the acute phase of pain (Figure 1C) and a partial reversal of mechanical hypernociception in the chronic phase (Figure 1D). After the last administration of CTX:SBA-15, a significant inhibition of mechanical hypernociception was still observed, lasting up to 10 days in the acute phase (Figure 1C) and for 17 days in the chronic phase (Figure 1D). This result differed from that of the CTX treatment, which did not present differences from PSNL group treated with PBS two days after the last administrations in both acute (Figure 1C) and chronic phases (Figure 1D). Interestingly, the antinociceptive effect of both CTX and CTX:SBA-15 was also observed 24 h after each administration, but it was more intense in the CTX:SBA-15 group when compared to treatment with CTX, in both phases (Figure 1E,F).

### 2.3. CTX:SBA-15 Complex Does Not Alter CTX Analgesic Mechanisms of Action Related to the Involvement of Formyl Peptide, α2-Adrenergic and Muscarinic Receptors

**Figure 2:**
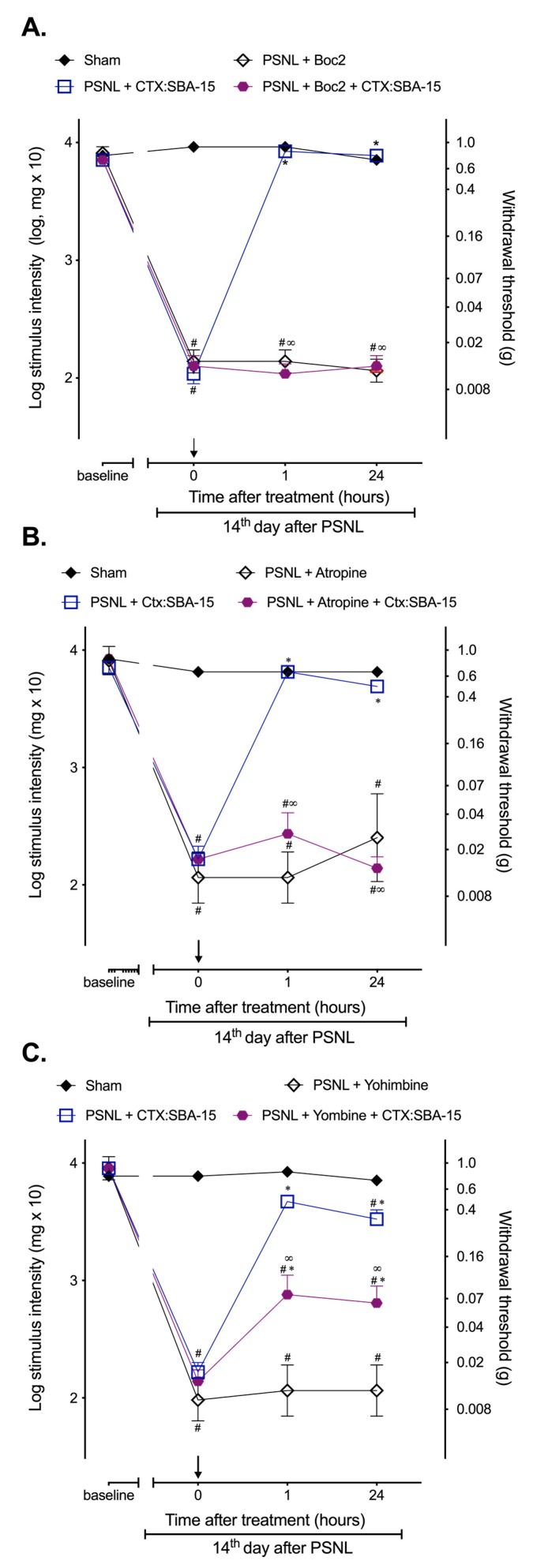
Pharmacological investigation of the CTX:SBA-15 antinociceptive mechanism of action. Animals were treated (s.c.) on the 14th day after PSNL with CTX:SBA-15 complex 30 min after administration of antagonists Boc2 (10 μg/kg, i.p.) (**A**), atropine (10 mg/kg, i.p.) (**B**), or yohimbine (2 mg/kg, i.p.) (**C**). Nociceptive threshold was assessed using the von Frey filaments before (baseline) and after PSNL surgery. Arrow indicates the time of CTX and CTX:SBA-15 administration. The results are expressed as the mean (± SEM) of n = 6. * *p* < 0.01 indicates significant difference when compared to the PSNL + antagonist group. ^#^ *p* < 0.01 indicates significant difference when compared to the Sham group. ∞ *p* < 0.01 indicates a statistically significant difference when compared to PSNL + CTX:SBA-15 group.

In the chronic period of pain (14 days after surgery), the animals submitted to PSNL were treated with specific receptor antagonist, 30 min prior CTX or CTX:SBA-15 administration, to investigate whether the conjugation to SBA-15 would modify previously known CTX mechanisms of action. Results demonstrated that Boc2 (formyl peptide receptors antagonist) (Figure 2A) or atropine (α2-adrenergic receptors antagonist) (Figure 2B) completely reversed the antinociception induced by CTX:SBA-15 complex 1 and 24 h after the treatment, while yohimbine (muscarinic receptor antagonist) (Figure 2C) partially reversed this effect, confirming the involvement of these receptors in the antinociceptive effect induced by CTX:SBA-15.

### 2.4. CTX Orally Administered Only Presents Analgesic Activity When Adsorbed to SBA-15

**Figure 3:**
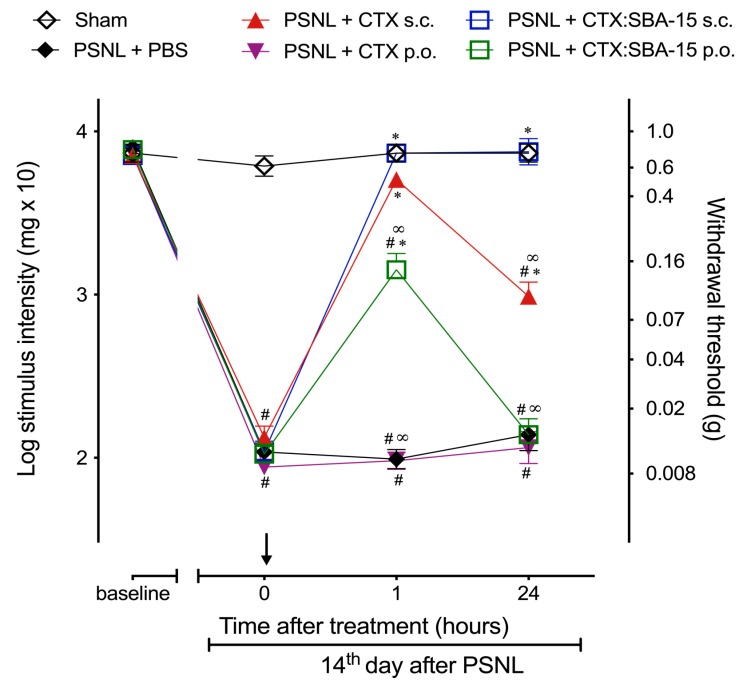
Oral treatment with CTX:SBA-15 reduces the mechanical hypernociception induced by PSNL model. Mice were submitted to PSNL surgery and on the 14th day treated, orally (p.o.) or subcutaneously (s.c.), with a single dose of CTX (40 μg/kg) or CTX:SBA-15 (54 μg/kg). The nociceptive threshold was assessed using the von Frey filaments. Arrow indicates the time of compounds administration. Results are expressed as mean (± SEM) n = 6, representative of two independent experiments. * *p* < 0.01 indicates significant difference when compared to the PSNL + PBS group. ^#^ *p* < 0.01 indicates significant difference when compared to the Sham group. ∞ *p* < 0.01 indicates a statistically significant difference when compared to PSNL + CTX:SBA-15 group.

Considering the fact that CTX has no effect when orally administered [27], and SBA-15, as a carrier, has physicochemical characteristics that may prevent the degradation of compounds by the gastric acid and its enzymes [22], it was next investigated whether the CTX:SBA-15 complex would exert antinociceptive effect upon PSNL injury when administered orally. Results demonstrated that unconjugated CTX, orally administered, has no antinociceptive effect in the chronic phase (14th day after surgery) of PSNL when compared to CTX administered subcutaneously (Figure 3). Surprisingly, orally administered CTX:SBA-15 induced partial reversal of mechanical hypernociception, observed 1 h after administration, even yet the effect was not as lasting as the effect observed by subcutaneous treatment (Figure 3).

### 2.5. Both CTX and CTX:SBA-15 Treatments Reduce Iba-1 and GFAP Spinal Cord Expression Induced by PSNL

**Figure 4:**
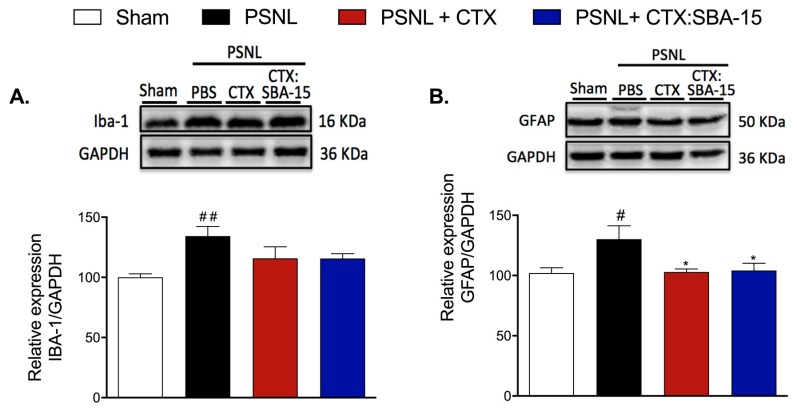
Effect of CTX and CTX:SBA-15 on the expression of glia cells markers after PSNL. Evaluation of Iba-1 (microglia/macrophages marker) (**A**) and GFAP (astrocytes marker) (**B**) expression were evaluated by Western blotting assay. Groups of animals were submitted to PSNL surgery and treated with 5 consecutive daily doses of CTX (40 μg/kg, s.c.) or CTX: SBA-15 (54 μg/kg, s.c.) from 0 to 4th day after PSNL. The dorsal horn of the spinal cord (ipsilateral) was collected on the 10th day after surgery. Results are expressed as mean (± SEM) n = 5–6 animals/group, representative of three independent experiments. ^#^ *p* <0.05 and ^##^ *p* < 0.01 indicates a statistically significant difference when compared to the Sham group. * *p* < 0.05 indicates statistically significant difference when compared to PSNL group.

Considering the relevance of microglia and astrocytes for the development of neuropathic pain after nerve injury [28], we next evaluated the expression of Iba-1 (ionized calcium binding adaptor molecule 1, microglia/macrophage marker) and GFAP (glial fibrillary acidic protein, astrocyte marker) in the spinal cord of PSNL groups (10 days after surgery). Results demonstrated that the PSNL group presented a significant increase in the expression of both Iba-1 and GFAP when compared to the control group (Sham) (Figure 4A,B). However, even though no differences in Iba-1 expression were observed in animals treated with CTX or CTX:SBA-15 complex when compared to PSNL animals, there was a significant difference in the variance amongst groups (F(3, 17) = 4.427; P = 0.0179) (Figure 4A). Concerning GFAP expression, both CTX and CTX:SBA-15 induced a reduction to basal levels in PSNL-induced GFAP expression (Figure 4B).

### 2.6. Both CTX and CTX:SBA-15 Modulate the Production and Release of Cytokines Induced by PSNL Model

**Figure 5:**
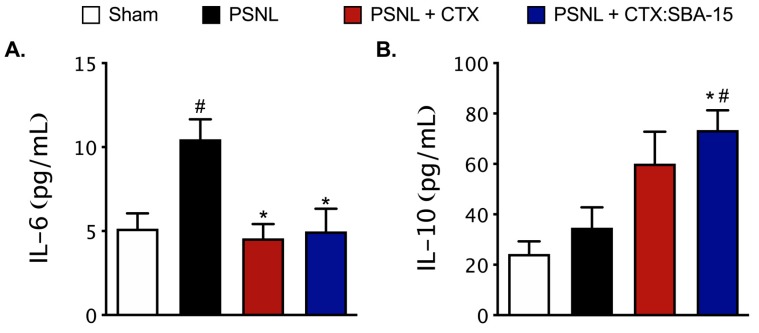
Effect of CTX and CTX:SBA-15, in the acute phase, on the release of cytokines in animals submitted to PSNL. Evaluation of IL-6 (**A**) and IL-10 (**B**) cytokines expression was performed by MULTIPLEX assay. The animals were submitted to PSNL surgery and treated with 5 consecutive doses of CTX (40 μg/kg, s.c.) or CTX: SBA-15 (54 μg/kg, s.c.) from 0 to 4th day after PSNL. The dorsal horn of spinal cord (ipsilateral) was collected on the 7th day after surgery. Results are expressed as mean (± SEM) n = 4–5 animals/group, performed in duplicate. ^#^ *p* < 0.05 indicates a statistically significant difference when compared to the Sham group. * *p* < 0.05 indicates statistically significant difference when compared to PSNL group.

Considering that spinal cytokines can modulate nociceptive behavior upon nerve injury [1], it was next evaluated the interference of both CTX and CTX:SBA-15 treatments on PSNL-induced cytokines release in the spinal cord 7 days after surgery. Results demonstrated that the IL-6 spinal levels were increased in PSNL animals and reduced to basal levels in both treatments with CTX and CTX:SBA-15 groups (Figure 5A). In relation to IL-10, an up-regulation of this cytokine was observed in the CTX:SBA-15 group, when compare to Sham or PSNL animals, despite the significant difference amongst groups’ variances (F (3.12) = 6.625; *p* = 0.0069) (Figure 5B). At the time period evaluated, there was no detection of IL-1β and TNF-α spinal levels (lower than detection limit) in studied groups (data not shown).

### 2.7. Antibodies Produced after CTX:SBA-15 Immunization Protect Against CTX Lethality and Do Not Affect CTX or CTX:SBA-15 Analgesic Effect

Given the immunogenicity of CTX and the increase in antibody production induced by antigen-SBA-15 conjugation, it was next investigated whether CTX conjugated to SBA-15 would have its antibody production increased. It was verified that after immunization with unconjugated CTX, CTX:SBA-15 or CTX:Al(OH)_3_, H_III_ mice (mouse line genetically selected for high antibody responsiveness) did not present significant expression of anti-CTX antibodies when compared to the controls (naïve and SBA-15 groups) (Figure S2A and Table 1). However, after the second dose, the serum of animals immunized with CTX showed a significant titer increase, that was even more augmented in serum from CTX:SBA-15 animals (Figure S2B and Table 1). It is important to point out that although CTX:SBA-15 immunization induced higher antibody titer compared to CTX group, this titer was lower when compared to the group immunized with CTX:Al(OH)_3_, used as a positive control.

**Figure 6:**
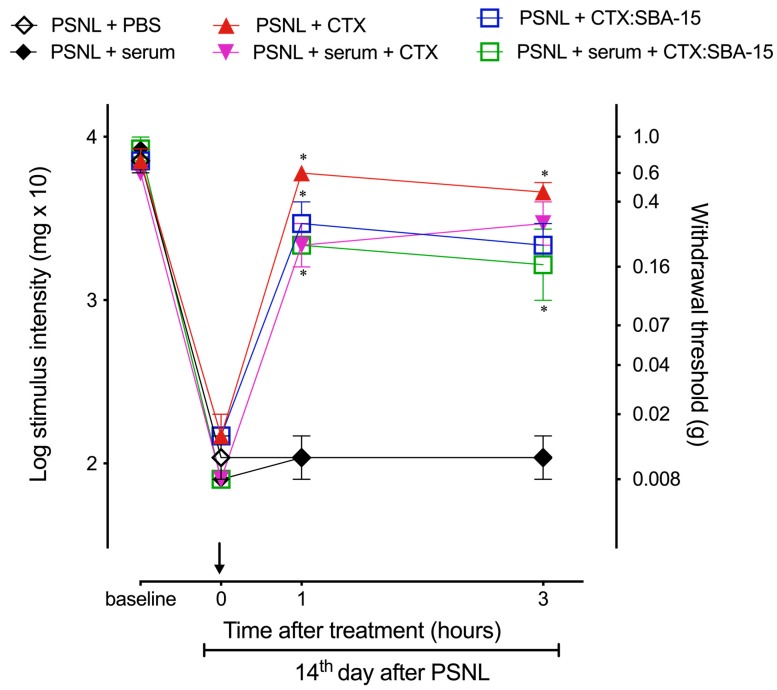
Anti-CTX antibodies do not affect antinociceptive effect of CTX and CTX:SBA-15 after PSNL. Groups of animals were treated on 14th day after PSNL with CTX (40 μg/kg, s.c.) or CTX:SBA-15 (54 μg/kg, s.c.) 15 min after administration of serum containing anti-CTX antibodies produced after immunization with CTX-SBA-15. The nociceptive threshold was assessed one and three hours after treatments using the von Frey filaments. Arrow indicates the time of compounds administration. The results are expressed as the mean of (± SEM) n = 5. * *p* < 0.001 indicates a statistically significant difference when compared to PSNL group.

Considering this increase in antibody titer, we next verified whether these antibodies would lead to resistance to the antinociceptive effect of both CTX and CTX:SBA-15, and to a protection against CTX lethality. Serum from H_III_ animals previously treated with CTX:SBA-15 (1:4, titer: 1024, 150 µl, i.v.) was injected in mice 15 min before CTX on a lethal dose (300 µg/kg). This passive immunization protected 75% of the animals, while all animals that did not receive a serum injection died within 24 h after the administration of the lethal dose of CTX. Although anti-CTX antibodies decreased CTX-induced lethality, even in a concentration 4 times higher than the one used in the lethality experiment (titer: 4096, 150 µl, i.v.), they did not interfere with the antinociceptive effect of both CTX and CTX:SBA-15 in PSNL model, since there was no significant difference in the antinociception induced by these treatments among the animals that received or did not receive the serum (Figure 6).

## 3. Discussion

Animal venoms and toxins have been widely described for their potential use as therapeutics for the treatment of a variety of diseases [29,30,31]. CTX is the main compound responsible for the high toxicity of *Crotalus durissus terrificus* venom [14], the toxin is capable of inducing antinociceptive and immunomodulatory effects [16,32,33]. However, its toxicity is a limiting factor to its potential therapeutic uses. It is known that SBA-15 silica, when used as a carrier, can offer a functional system as a gatekeeper to control release, allowing a drug or other compounds to reach their target in a suitable concentration, and due to the physical properties of mesoporous silica nanoparticles (MSNs), it may be possible to achieve a drug loading of about 30% above the primary dose [26]. In this study, it was investigated whether SBA-15 could decrease CTX toxicity, maintaining or enhancing its analgesic effect.

The SBA-15 silica is an inorganic substance that possesses uniform hexagonal nanostructured mesopores with pore diameter between 10 and 50 nm; it is capable of interacting with distinct proteins/peptides in its interior (encapsulation) and/or surface (adsorption), protecting them against proteolysis, organic solvents, high shear stresses, and low pH [22,34,35]. As such, it can be considered a suitable vehicle able to carry, protect, and deliver the encapsulated/adsorbed molecules [22].

Studies report wide interfamily, intergender, interspecies, and intraspecies variability that exists among animal venoms. Regarding interspecies variation, this may still occur individually, for several reasons, including seasonal variation, diet, habitat, age, and sexual dimorphism [36,37,38]. Considering the variability of each lot, due to the pool constitution of venoms, the LD_50_ was evaluated, not only for CTX:SBA-15 complex, but also for CTX used throughout this project. Results showed that, when complexed to silica SBA-15, CTX requires a dose 35% higher than the group treated with CTX by itself to induce the lethal effect in 50% of the animals in the group. Although this average value was within the confidence interval of unconjugated CTX, the upper margin showed an increase of 63%, shifting the confidence limit. In this way, these results corroborate the data concerning the decrease of toxicity to bacterial toxins induced by SBA-15 (Sant'Anna OA, unpublished data). Also, they are in accordance with recently published data showing that MSNs are capable of reducing toxicity of anticancer drugs in the mice model [25].

The antinociceptive effect of CTX was previously described [16,39]; nevertheless, the toxicity of the compound is a limiting factor for increasing dosages or for extended treatment periods, especially considering chronic or debilitating circumstances. Therefore, the PSNL-induced neuropathic pain model [40] was used to evaluate the antinociceptive effect of CTX conjugated to silica SBA-15 compared to CTX by itself. In this model, several neurochemical and protein changes occur in the dorsal root ganglion and spinal cord. These changes may result in development of symptoms of neuropathic pain, including hypernociception to mechanical stimuli [40,41]—where the pain observed is characterized by a first typically inflammatory and easily controlled phase, from 0 until 7 days, followed by a neuropathic phase, observed after 10 or 14 days, which is currently poorly controlled by clinically available therapeutic drugs [4,10,41].

Both CTX and CTX:SBA-15 induced reversion of mechanical hypernociception induced by PSNL, in both acute and chronic phases of pain. However, the effect of CTX:SBA-15 persists longer than that observed only with CTX. In addition, 24 h after CTX:SBA-15 administration, the animals still showed complete reversion of hypernociception, while animals treated with CTX showed only partial reversal of pain.

The analgesic effect of CTX on neuropathic pain was previously demonstrated in a model of total sciatic nerve transection. In this model, when topically applied to the site of transection, CTX prevents the development of hypernociception induced by surgery for at least 60 days, while when subcutaneously applied this antinociceptive effect was observed for only 2 h, no longer being observed 24 h later [16].

Recent studies have shown that MSNs is a potential controlled drug delivery system [26,42,43]. Based on our data, it can be suggested that this long-lasting effect of CTX complexed to SBA-15 silica could be associated with a controlled release of the toxin, which would explain not only the prolonged antinociceptive effect but also the partial reversal observed one hour after its administration.

According to the literature, the anti-inflammatory effect of CTX can be mediated by formyl peptide receptors [13,44]. The formyl peptide receptors are known to be the targets of lipoxin A4 and annexin. Lipoxin A4 is a lipid mediator produced from arachidonic acid via the lipoxygenases pathway; it has high affinity binding for G protein-coupled receptors and thus stimulates phagocytosis by monocytes and macrophages and decreases cytokine release, facilitating inflammation resolution [45]. Moreover, in the neuropathic pain model induced by neurectomy, the lipoxygenase pathway is involved in the antinociceptive effect of CTX [16]. Lipoxins have a role in the processing of pain through the communication between the immune system and the sensory nervous system [45]. In the PSNL model, the antagonist of these receptors blocks the antinociceptive effect induced by the CTX:SBA-15 complex in chronic phases of the pain, confirming that silica does not alter CTX mechanism of action.

The processing of pain is a complex mechanism that integrates a series of structures from the periphery to the CNS, via the spinal cord to higher cerebral structures; at the dorsal horn of the spinal cord, pain can be modulated by several mechanisms, among them cholinergic inhibitory interneurons and descending pathways, such as serotoninergic and noradrenergic systems [1]. The antinociceptive effect induced by CTX in another neuropathic pain model involves the muscarinic and α-adrenergic receptors [16]. In the present work, it was observed that these receptors mediate the antinociceptive effect of CTX:SBA-15, as well as previously reported for CTX. Taken together, these results confirm the participation of formyl peptide, muscarinic, and noradrenergic receptors in the analgesic effect of the complex CTX:SBA-15 and also demonstrate that silica SBA-15, while potentiating the antinociceptive effect of CTX, does not change its mechanism of action.

As previously described, silica SBA-15 presents physicochemical properties that may protect different compounds from possible degradation by digestive acids and enzymes. This property allows for the oral administration of some antigens, such as hepatitis B vaccine, which before the conjugation had no effect [46]. It is known that CTX when orally administrated has no effect, since as a protein, it has low oral bioavailability [47]. Our results show that the oral treatment with CTX:SBA-15, different from CTX per se, promotes analgesic effect in mice submitted to PSNL surgery, as occurring through the subcutaneous route, thus enabling a better route of administration for CTX and further expanding the use of SBA-15 as a carrier for drugs that cannot be administered orally.

Previous studies have suggested that immune cells of the nervous system, such as inflammatory cells and glial cells, play an important role in the pathogenesis of hypersensitivity after peripheral nerve injury [28]. In the spinal cord, there is an increase in the activation of glial cells, especially astrocytes and microglia [48,49], suggesting that the control of this activation may be a target for drug development. Treatment with CTX or CTX: SBA-15 reduces the expression of IBA-1 and GFAP, suggesting a reduction in glial cell activity after treatment. Increased activity and number of glial cells may contribute to neuropathic pain, since activation of glial cells due to neuropathy observed after peripheral nerve damage leads to the release of cytokines, such as TNF-α, IL-6, IL-10, and IL-1β and chemokines at the CNS [50,51], which can modulate inflammatory response and nociceptive transmission [1,52]. Interleukins contribute to cortical mechanisms of sensitization [1], and in particular IL-6 may have an important role in the development and maintenance of pathological pain, such as neuropathic pain [53,54]. Neuropathic models, including PSNL, have been shown to enhance expression of IL-6 in the spinal cord and dorsal root ganglia [55,56]. Additionally, IL-6 has demonstrated a crucial role in nociceptive plasticity [57,58], contributing to peripheral and central sensitization [55]. Hence, IL-6 is a potential target for the development of therapeutic drugs for neuropathic pain, as shown in recent reports using experimental and clinical models of chronic pain [59,60].

Likewise, proinflammatory cytokines have an important role in the development and maintenance of chronic pain (pronociceptive process), anti-inflammatory cytokines also modulate the antinociceptive process inhibiting the production of proinflammatory cytokines [1]. Among these cytokines, IL-10 has been described as a potent anti-inflammatory cytokine and an important modulator of neuropathic pain. IL-10 attenuates proinflammatory cytokines synthesis, via kappa B factor [52]. Gene therapy with IL-10 in animal models of neuropathic pain reduce the symptoms of mechanical hypernociception [61,62].

In addition, a recent report showed that animals with spine nerve ligation (SNL) treated with C-glycosyl compound, (a drug with similar mechanism to gabapentin which is a first-line medication for chronic neuropathic pain treatment), had their IL-10 expression increased [63].

Previous reports showed that in an animal model of colitis, mice treated with CTX present an up regulation of local anti-inflammatory cytokines, such as TGF-β and IL-10, and a decreased expression of proinflammatory cytokines, such as IL-6 and TNF-α [64]. In this study, mice submitted to PSNL surgery and treated with unconjugated CTX and CTX:SBA-15 showed a down regulation of IL-6 and an increase of IL-10 protein expression, suggesting a modulation in cytokines production that might be contributing to the anti-inflammatory and antinociceptive properties of CTX.

The administration of silica particles consistently improves antibody response [65,66], therefore the production of anti-CTX antibodies were evaluated. The titers of anti-CTX in serum after the second dose was two times higher in the group immunized with the complex CTX:SBA-15 when compared to CTX. This ability of silica SBA-15 to increase antibody production could be due to the fact that encapsulation/absorption in SBA-15 could assure a better epitope recognition of the antigen by the antigens presenting cells (APC), promoting a successful activation of the immune system [22]. In addition, the increased numbers of T, and specially B lymphocytes observed in mice immunized with Hepatitis A vaccine or human gamma-globulin adsorbed on SBA-15, associated to the high titers of secretory antibodies, may indicate that the use of SBA-15 as an adjuvant is able to induce an increase in the proliferation and recruitment of immunocompetent cells [35], improving the immune response. However, in a chronic disease such as neuropathic pain, long-term treatment with repeated doses of medicines are necessary; in such cases, the increased antibody production is undesirable. In relation to this, our data showed that the anti-CTX antibodies produced by CTX:SBA-15 immunization, although protecting the organism against the lethal CTX effect, do not interfere with the antinociceptive effect of CTX.

## 4. Conclusions

These results confirm that the SBA-15 acts as a carrier and may protect the CTX toxin until reaching the target, as well as decreasing its toxic effect. Furthermore, when conjugated to SBA-15, the oral route becomes a possible means of CTX administration. The potential controlled release of the CTX may explain the long-lasting antinociceptive effect of the complex CTX:SBA-15, reducing the activity of glia cells and modulating the release of cytokines, not affecting the mechanism of action of CTX. In summary, this study extends the knowledge for the use of SBA-15 and also enables new formulation and new route of administration for crotoxin.

## 5. Materials and Methods

### 5.1. Animals

Experiments were performed using male C57BL/6 null mice weighting 18–22 g, aged 8–12 weeks, from the animal facility of Butantan Institute, and on male mouse line genetically selected for high antibody responsiveness (H_III_) of same weight and age from the Immunogenetic Laboratory of Butantan Institute. Mice were housed in numbers of 4 to 5 per cage and kept under a 12-hour light/dark cycle with food and water ad libitum. The room was kept under 21 ± 2.0 °C and at a humidity of 50% ± 10% RH. All behavioral tests were performed between 8:00 a.m. and 5:00 p.m. Experimental procedures were approved by the Ethics Committee on Animal Use of the Butantan Institute (CEUAIB, protocol number 1320/14, approved on 07/2015) and in accordance with the National Council of Animal Experimentation Control (CONCEA) regulation and with the International Association for the Study of Pain guidelines for the ethical use of conscious animals in pain research published by the International Association for the Study of Pain [67].

### 5.2. Crotoxin (CTX)

CTX was purified from a pool of lyophilized *Crotalus durissus terrificus* venom, containing several adult specimens of different age and gender, provided by the Laboratory of Herpetology, Butantan Institute. Purification was performed by anion-exchange chromatography as previously described [68,69] and was kindly supplied by Dr. Sandra Coccuzzo Sampaio Vessoni from Laboratory of Pathophysiology, Butantan Institute.

### 5.3. SBA-15 Synthesis

The SBA-15 synthesis and adsorption characterization were performed as previously reported [70]. The small-angle X-ray scattering (SAXS) characterization was performed [34] and silica SBA-15 was kindly supplied by Dr. Tereza S. Martins from Federal University of São Paulo.

### 5.4. Preparation of the Complex (CTX:SBA-15)

The CTX was diluted in phosphate buffered saline (PBS) pH 7.4, slowly added to the silica SBA-15 (1:10) and held for 24 h at 2–8 °C, with occasional stirring.

### 5.5. Drug Administration

In in vivo assays, except for the LD_50_ experiments_,_ where the doses will be presented in the test description, the following compounds were used: 40 μg/kg (in 200 µL of PBS) of CTX [13] or 54 μg/kg (in 200 µL of PBS) of CTX:SBA-15 administered by subcutaneous (s.c.) or oral route (p.o.); the formyl peptide receptor antagonist, Boc2 (butoxycarbonil-Phe-Leu-Phe-Leu-Phe, #Phoenix Pharmaceutical Inc, USA) at 10 μg/kg (in 100 µL of sterile saline), the muscarinic receptors antagonist, atropine sulphate (Sigma #5908996) at 10 mg/kg (in 100 µL of sterile saline), and the α-adrenergic receptor antagonist, yohimbine hydrochloride (Sigma #y3125) at 2 mg/kg (in 100 µL of sterile saline), were intraperitoneally (i.p.) administered. All the antagonists were administered 30 min prior the treatment with CTX or CTX:SBA-15.

### 5.6. Lethal Dose (LD_50_) Determination

Null animals (n = 6/group) received unconjugated CTX, diluted in PBS, at concentrations of 600, 300, 150, 75, 37.5, or 18.75 μg/kg administered (100 μL, i.p.) (doses were selected based on CTX LD_50_ previously established [71]). For the determination of the LD_50_ of the complex (CTX:SBA-15), the LD_50_ of unconjugated CTX was consider as intermediate dose; therefore, CTX:SBA-15 complex was injected at 424, 212, 106, 53, or 26.5 μg/kg doses (100 μL, i.p.). LD_50_ were calculated using the Probit analysis method [72].

In a different experiment, to verify whether serum antibodies produced against CTX:SBA-15 could affect lethality of animals injected with unconjugated CTX, naïve animals were injected with the serum-diluted 1:4 (titer: 1024, 150 µl, i.v.), and after 15 min treated with a lethal dose of CTX (300 µg/kg, i.p.). Animals’ survival was monitored up to 48 h after injection; LD_50_ was calculated using the Probit as described above.

### 5.7. General Experimental Design

Considering the diversity of venoms constituting each pool, the lethal dose 50% (LD_50_) was determined for the current lot of CTX, unconjugated or complexed with silica SBA-15 (CTX:SBA-15), in order to determine if CTX would have its toxicity decreased by the silica SBA-15. Based on the LD_50_ obtained for both CTX and CTX:SBA-15, we determined the doses used throughout this work—40 μg/kg of CTX, a known non-toxic dose [13] or 54 μg/kg of CTX:SBA-15 (a dose 35% higher when compared to CTX, based on the LD_50_ test). Next, it was determined the antinociceptive effect of unconjugated CTX and CTX:SBA-15 complex (s.c.) on the acute or chronic phases of hypernociception induced by PSNL, administered in 1 (4th day or 14th day after surgery) or 5 doses (0 to 4th day or 14th to 18th day after surgery, 1 daily dose for 5 consecutive days). To evaluate whether SBA-15 silica would change the mechanism of action previously described for CTX in the literature, the animals were submitted to PSNL and in chronic phase (14th day after surgery) were treated with specific receptor antagonists for formyl peptide, α2-adrenergic, and muscarinic receptors before treatment with CTX:SBA-15. In addition, to determine the protective effect of SBA-15 to CTX, both CTX and CTX:SBA-15 were administered by oral route (1 dose at 4th day after surgery).

Spinal cord microglia and astrocytes expression were determined on the 10th day after surgery, in the group treatment with 5 doses of CTX or CTX:SBA-15 in the acute phases (one daily dose for 5 days, starting on day 0–day of the surgery). The interference of CTX:SBA-15 on the IL1β, IL10, IL6, and TNFα levels was determined at the dorsal horn of the spinal cord on the 7th day after surgery by xMap method (MULTIPLEX).

Finally, since the unconjugated CTX is immunogenic and the silica SBA-15 may increase the production of antibodies, it was necessary to evaluate the production of anti-CTX antibodies after immunization and the participation of these antibodies in both lethal and analgesic activities. For the analysis of the immunogenicity of the compound, H_III_ mouse line genetically selected according to phenotype for high antibody responsiveness to immunogens [73,74] was used.

Considering that in a long-term treatment these antibodies could affect the effect of CTX, to investigate the interference of the antibody production to the antinociception, a passive immunization was performed on the 14th day after PSNL by injecting serum obtained from the H_III_ mice previously injected with CTX:SBA-15 (titer: 4096, 150 µl, i.v.) and 15 min after the animals were treated with CTX or CTX:SBA-15. In order to confirm the neutralizing potency of the serum containing anti-CTX antibodies, naïve animals were injected with the serum-diluted 1:4 (titer: 1024, 150 µl, i.v.), and 15 min after were treated with a lethal dose of CTX (300 µg/kg), intraperitoneally. Survival was monitored up to 48 h after injection.

### 5.8. Neuropathic Pain Model—Partial Sciatic Nerve Ligation (PSNL)

Unilateral PSNL was performed according to the method previously described [40]. Briefly, mice were anaesthetized with isoflurane (1.5% in oxygen) and the left sciatic nerve was exposed at the mid-thigh level. A partial tying was made up of the sciatic nerve by tightly ligating 1/3 to 1/2 of the medial dorsal nerve’s diameter using fine silk (8-0, Bioline, BR). Then, silk sutures (5-0, Bioline, BR) were used to suture the incision. For the sham-operation (control group), mice underwent the same procedure without ligation of the nerve.

### 5.9. Behavioural Test—Mechanical Hypernociception Assessment Using von Frey Filaments

Mice were placed individually in bottomless plastic cages on an elevated wire grid to allow access to the paws and habituated to the experimental environment (room and apparatus) for 20 min before test. Mechanical hypernociception was assessed using von Frey hair filaments by measuring the paw withdrawal nociceptive threshold, taken as the lowest force that evoked a brisk paw withdrawal response to one of five repetitive stimuli [75]. Briefly, the plantar surface of the right hind paw was stimulated with von Frey hairs, a logarithmic series of calibrated monofilaments in a series of ascending force, ranging from 0.903 (8 mg) to 3.0 (1000 mg). The log of the hairs is determined by log10 (milligrams) and the mechanical threshold is represented as previously described [76].

### 5.10. Glia Cell Expression Evaluated by Western Blotting

The dissection and collection of the dorsal horn from the spinal cord tissue from ipsilateral lumbar segments (L3–L6) was performed on the 10th day after surgery. Contents were homogenized and lysed in buffer containing Hepes-NaOH (1 M, pH 7,9), NaCI (1,54 M), EGTA (200 mM), Triton-X 100 (1%), cocktail protease inhibitor and phosphatase (1:300, Sigma-Aldrich, USA). Protein samples (30 µg) were separated by electrophoresis on acrylamide gel SDS-PAGE (8% and 10%) and transferred to nitrocellulose membranes. Incubation consisted of one hour at room temperature with blocking solution containing 5% blocking agent (GE Healthcare Life Science, Pittsburgh, PA, USA) dissolved in Tris-Buffered Saline–10% Tween 20 (TBS-T) followed by incubation with primary antibodies [anti-IBA-1, 1:500 (Abcam # ab178847), anti-GFAP, 1:5000 (Abcam, #ab53554), and anti-GAPDH (1:5000, Abcam # ab8245) as loading control] overnight at 4 °C in TBS-T/5% bovine serum albumin (BSA). Then, membranes were washed 3 times for 5 min in TBS-T and incubated for one hour with secondary antibodies conjugated with peroxidase at 1:5000 in 5% blocking agent (GE Healthcare Life Science, Pittsburgh, PA, USA) dissolved in TBS-T. Bands were visualized using ECL (Pierce) solution with digital image capture system and an optical density was determined by image lab software (UVITEC Cambridge, version 15.03b, England, UK).

### 5.11. Cytokine Level Evaluation

The spinal cord was dissected, and the dorsal horn was collected from the region comprised between lumbar segments (L4–L6), ipsilateral to the nerve injury. The tissue was homogenized and lysed in buffer containing PBS (0.01 M, pH 7.4), Tween 100 (0.1%), and cocktail protease inhibitor and phosphatase (1:300, Sigma-Aldrich, USA). The homogenate was centrifuged at 10,000 × *g* for 5 min at 4 °C. An aliquot of the supernatant was separated for determination of proteins content by Coomassie colorimetric method (Bradford Reagent #23238–ThermoFisher Scientific, USA) [77,78]. The samples were normalized (protein concentration 1.2 µg/µL) and the cytokines concentrations were determined by xMap method (MULTIPLEX), using the commercial kit (# MCYTOMAG-70K-04 - IL1β, IL10, IL6 e TNFα, Millipore, USA). All samples were analyzed in duplicate and the reading was performed by the equipment Luminex 200–Software xPonent/Analyst version 4.2 (LEAC lab, Sao Paulo, Brazil).

### 5.12. Anti-CTX Antibody Titration by ELISA and Passive Immunization

For this test, the H_III_ mouse line was used. Animals were divided into 5 groups of immunization (s.c.): Naive, SBA-15 (486 µg/kg), CTX (40 µg/kg), CTX:SBA-15 (54 µg/kg), and CTX:Al(OH)_3_ (54 µg/kg). Booster dose was given after 30 days of immunization. Blood was collected from the retro-orbital plexus 15 days after the first immunization and 20 days after the second dose (booster). Titers of anti-CTX antibodies were determined by ELISA, as described previously [79]. Briefly, microplates were coated with 1 µg CTX per well, and titration was determined as the reciprocal of the highest dilution that showed an absorbance at 450 nm greater than 0.050.

To investigate the interference of anti-CTX antibodies produced by H_III_ mice on the antinociception induced by CTX:SBA-15 on C57Bl/6 submitted to PSNL model, a passive immunization was performed. On the 14th day after PSNL, serum obtained from H_III_ mice previously injected with CTX:SBA-15 (titer: 4096, 150 µl, i.v.) was injected and 15 min later the animals were treated with CTX or CTX:SBA-15. Nociceptive behavior was measured by von Frey hairs 1 and 3 h after treatments.

### 5.13. Data Analysis

Statistical analysis of behavioral data was carried out using two-way analysis of variance (ANOVA) followed by a Turkey’s post-test. For the biomolecular assays, one-way ANOVA followed by Turkey’s t test. Statistical significance was set at *p* < 0.05. These evaluations were performed using the statistics package Prism software (Graph Pad Software, version 6.0, La Jolla, CA, USA).

## Figures and Tables

**Table 1:** Titration of anti-CTX antibodies in H_III_ mice. Results are expressed as the mean (± SEM) n = 4–5.

Titration of Antibodies		
Groups	15 days after first dose	20 days after second dose
Naive	256	256
SBA-15	256	128
CTX	256	1024
CTX:SBA-15	256	4096
CTX:Al(OH)_3_	256	32,768

## Supplementary Materials

The following are available online at https://www.mdpi.com/2072-6651/11/12/679/s1, Table S1. Evaluation of lethality induced by CTX and CTX:SBA-15. The survival time of each animal was followed for 48 h after administration (i.p.) of CTX (600, 300, 150, 75, 37.5, and 18.75 µg/kg) or complex CTX:SBA-15 (424, 212, 106, 53, and 26.5 μg/kg), Figure S1. Effects of SBA-15 on mechanical hypernociception induced by PSNL. The pain threshold of the animals underwent surgery (sham and PSNL) was evaluated from day 0 (baseline) to 35th days after PSNL. Measurements were performed 1 h after a single administration of SBA-15 (486 µg/kg, s.c.) on the 3rd and 14th days after surgery. Arrows indicate the days of SBA-15 administration. The nociceptive threshold was assessed using the von Frey filaments. Results are expressed as mean (± SEM) n = 6, Figure S2. Titration of anti-CTX antibodies after administration of CTX and CTX:SBA-15 in H_III_ mice. Representative curves obtained after the first dose (A) and booster dose (B) of SBA-15 (486 μg/kg, s.c.), CTX (40 μg/kg, s.c.), CTX:SBA-15 (54 μg/kg, s.c.), or CTX:Al(OH)_3_ (54 µg/kg, s.c.). Results are expressed as the mean (± SEM) n = 4-5.
