# Supplementary material for: Crotoxin Conjugated to SBA-15 Nanostructured Mesoporous Silica Induces Long-Last Analgesic Effect in the Neuropathic Pain Model in Mice
Source: Toxins (Basel). 2019 Nov 20;11(12):679. doi: 10.3390/toxins11120679 (PMC6949982; doi:10.3390/toxins11120679)
Supplement: Supplementary file 1 [file toxins-11-00679-s001.pdf]

Supplementary Materials: Crotoxin Conjugated to SBA-15 Nanostructured Mesoporous Silica Induces Long-Last Analgesic Effect in the Neuropathic Pain Model in Mice

Morena Brazil Sant'Anna, Flavia Souza Ribeiro Lopes, Louise Faggionato Kimura, Aline Carolina Giardini, Osvaldo Augusto Sant'Anna and Gisele Picolo

Table S1. Evaluation of lethality induced by CTX and CTX:SBA-15**.** The survival time of each animal was followed for 48 hours after administration (i.p.) of CTX (600, 300, 150, 75, 37.5 and 18.75 μg/kg) or complex CTX:SBA-15 (424, 212, 106, 53 and 26.5 μg/kg).

**Compound**	**LD_{50} (μg/kg)**	**95% Confidence Intervals (μg/kg)**
CTX	106	63.2–177.9
CTX:SBA-15	143.2	70.4–291.2

CTX- Crotoxin; CTX:SBA-15- CTX absorbed to SBA-15.

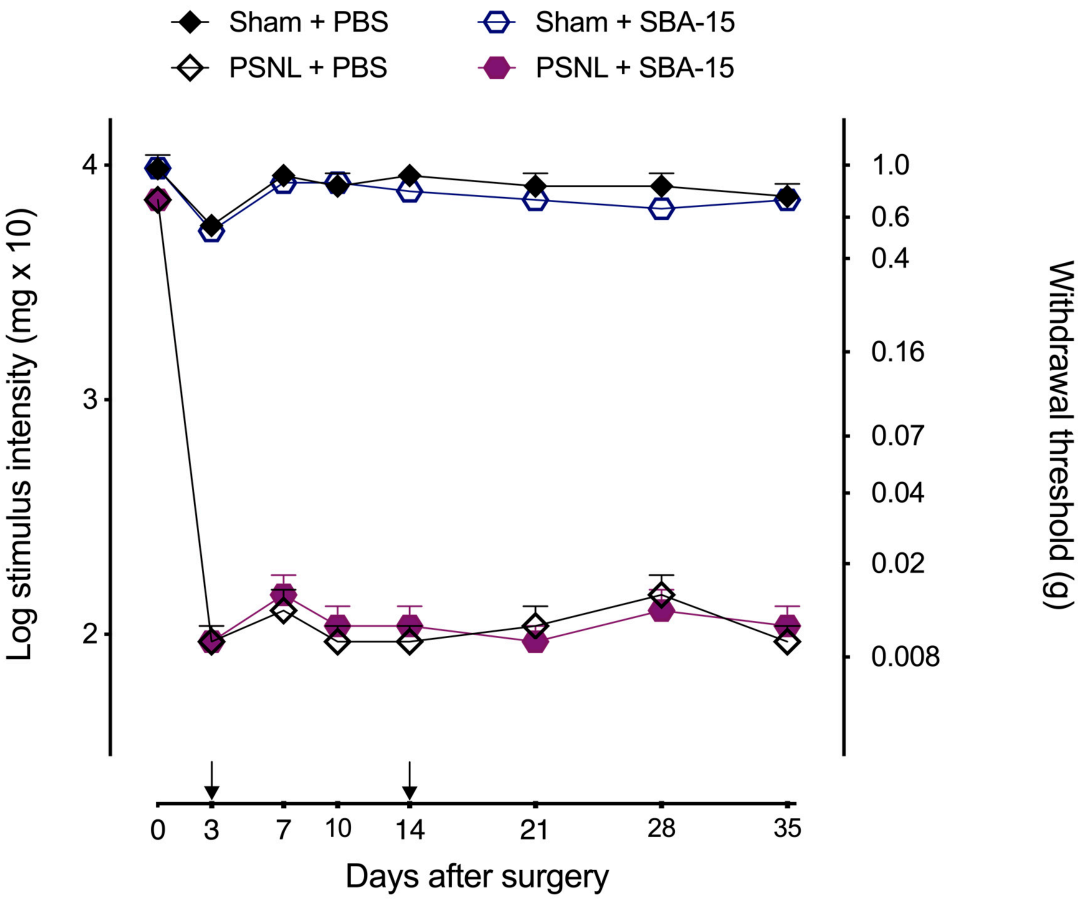

Figure S1. Effects of SBA-15 on mechanical hypernociception induced by PSNL. The pain threshold of the animals underwent surgery (sham and PSNL) was evaluated from day 0 (baseline) to 35th days after PSNL. Measurements were performed 1 hour after a single administration of SBA-15 (486 μg/kg, s.c.) on the 3rd

and 14th days after surgery. Arrows indicate the days of SBA-15 administration. The nociceptive threshold was assessed using the von Frey filaments. Results are expressed as mean (± SEM) n = 6.

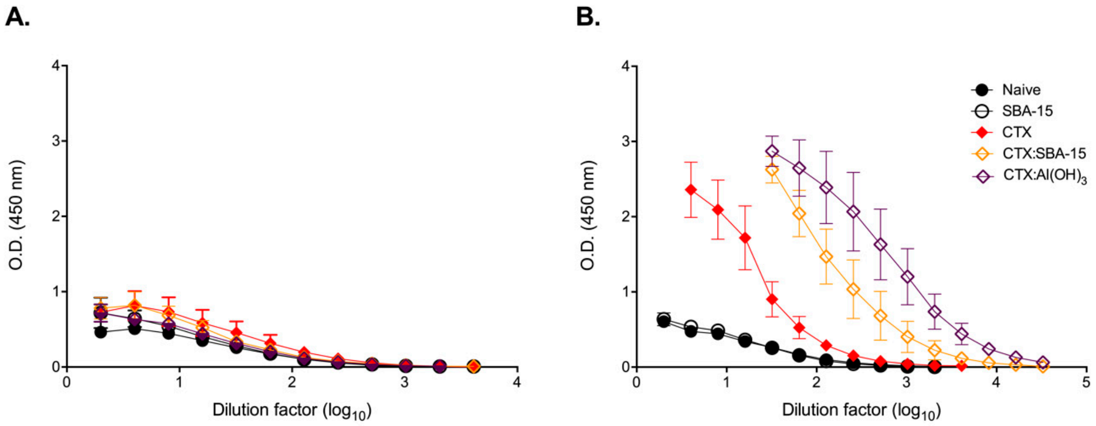

Figure S2. Titration of anti-CTX antibodies after administration of CTX and CTX:SBA-15 in H$_{III}$ mice. Representative curves obtained after the first dose (A) and booster dose (B) of SBA-15 (486 µg/kg, s.c.), CTX (40 µg/kg, s.c.), CTX:SBA-15 (54 µg/kg, s.c.) or CTX:Al(OH)$_3$ (54 µg/kg, s.c.). Results are expressed as the mean (± SEM) n = 4–5.
